# Esophageal Contractions After Wet and Dry Swallows in Patients With Esophagitis, Chagas' Disease and Idiopathic Achalasia

**DOI:** 10.4021/gr223w

**Published:** 2010-07-20

**Authors:** Jucileia Dalmazo, Roberto Oliveira Dantas

**Affiliations:** a Department of Medicine, Medical School of Ribeirao Preto, University of Sao Paulo, Ribeirao Preto, SP, Brazil

**Keywords:** Esophageal contractions, Idiopathic achalasia, Chagas’ disease, Esophagitis

## Abstract

**Background:**

In normal subjects the distal esophageal response to dry swallows differs from that of wet swallows. Our aim in this investigation was to compare the esophageal contractions of the proximal and distal esophageal body to wet and dry swallows.

**Methods:**

We studied the esophageal contractions of eight patients with idiopathic achalasia, 37 patients with Chagas’ disease, 28 patients with esophagitis, and 31 normal volunteers using manometric examination with continuous perfusion. The esophageal contractions were measured at 2 cm (proximal) and 22 cm (distal) from the upper esophageal sphincter. Five swallows of a 5 ml bolus of water alternated with 5 dry swallows were performed.

**Results:**

In the proximal esophagus there was no difference between wet and dry swallows. In patients with esophagitis and volunteers the contractions in the distal esophagus had greater amplitude with wet swallows than with dry swallows. Contraction amplitude was lower than the amplitude of the other groups, in both the proximal and distal esophageal body in achalasia, and in distal esophageal body in Chagas’ disease. The interval between the upstroke of contractions in the proximal and distal esophageal body was longer in volunteers and patients with esophagitis than in patients with Chagas’ disease and achalasia.

**Conclusions:**

Wet swallows cause higher amplitude of contraction in distal esophagus than dry swallows, which is not seen in diseases with impairment of esophageal innervation (achalasia and Chagas’ disease). In the proximal esophagus there is no difference in contractions caused by wet or dry swallows.

## Introduction

The esophageal response to swallows is a peristaltic contraction that crosses the entire esophageal body, from the upper esophageal sphincter (UES) to the lower esophageal sphincter (LES).

The esophageal contractions are regulated by the central nervous system and by the myenteric plexus [1]. They may change with the characteristics of the bolus swallowed. In distal esophageal body wet swallows cause esophageal contractions that differ from those caused by dry swallows [2-4].

In esophageal involvement by Chagas’ disease (secondary achalasia) and idiopathic achalasia (primary achalasia), there is a loss of the neurons of the esophageal myenteric plexus [5-8], which causes abnormalities of esophageal motility, i.e., absent or partial LES relaxation and aperistalsis in the esophageal body [6, 8-10]. These motility alterations are always observed in idiopathic achalasia because they are required for the diagnosis. Chagas’ disease causes a range of esophageal motility alterations from minor changes to achalasia [11, 12], because the loss of the myenteric neurons is not always intense [5].

Some patients with esophagitis may have alterations of distal esophageal motility [13]. Esophagitis may be associated with ineffective esophageal motility [14], although this result has not been confirmed by other investigations [15].

The esophageal innervation and muscles of the distal segment of the esophagus differ from those of the proximal segment [1, 16]. In the distal esophageal body of normal subjects wet swallows cause greater amplitude of esophageal contractions than dry swallows [2-4], a fact not observed in Chagas’ disease [17].

Our aim in this investigation was to evaluate the proximal and distal esophageal contractions after wet and dry swallows in patients with esophagitis, idiopathic achalasia, Chagas’ disease and normal volunteers. Our hypothesis was that there is no difference in esophageal response to wet and dry swallows in diseases that compromise the esophageal innervation (achalasia and Chagas’ disease).

## Patients and Methods

We studied 8 patients with idiopathic achalasia, 37 patients with Chagas’ disease, 28 patients with esophagitis, and 31 controls.

The patients with esophagitis were 8 men and 20 women aged 25 - 73 years (mean: 50.0 years). They complained of heartburn and acid regurgitation but did not have dysphagia. The endoscopic esophageal examination found grade A or B esophagitis according to the Los Angeles classification [18].

All patients with Chagas’ disease, 20 men and 17 women aged 23 - 78 years (mean: 58.3 years), complained of dysphagia and had a positive serologic test for the disease. Radiologic examination showed bolus retention and delayed bolus transit. Delayed esophageal bolus transit was seen when a 10 ml 100% barium sulfate bolus took more than 10 seconds to cross the entire esophageal body from UES to LES. In the manometric examination they had non-peristaltic contractions in the esophageal body in more than 70% of wet swallows and partial or absent LES relaxation. Non-peristaltic contractions were observed when there was a complete absence of motor activity after a wet swallow or when the onset of contractions occurred within less than one second between recordings separated by a distance of 5 cm. Incomplete LES relaxation occurred when the nadir of the sphincter pressure after a wet swallow did not decrease less than 5 mmHg above the intragastric pressure.

The patients with idiopathic achalasia were four men and four women aged 17 - 68 years (mean: 41.0 years). They complained of dysphagia, had a negative serologic test for Chagas’ disease, had not lived in areas where the disease was endemic, and radiologic esophageal examination showed that they had bolus retention and delayed bolus transit. In 100% of wet swallows the esophageal contractions were non-peristaltic and LES relaxation was partial or absent.

The control group consisted of normal volunteers, 10 men and 21 women aged 18 - 68 years (mean 43.7 years). They had no symptoms, and manometric examination showed that they had peristaltic contractions and complete LES relaxation in more than 90% of wet swallowing. All patients and volunteers gave written informed consent to participate in the study before they were submitted to manometric examination. The protocol of the examination was approved by the Human Research Ethics Committee of the University Hospital of Ribeirao Preto (SP), Brazil.

The manometric examination was performed with a round eight-lumen polyvinyl catheter with an outer diameter of 4.5 mm and an inner diameter of 0.8 mm (Arndorfer Specialities, Inc, Greendale, Wisconsin USA). The four proximal lateral openings of the catheter and the four distal lateral openings at the same level were spaced 5 cm apart. They were connected to external pressure transducers (PVB Medizintechnik Gmb H, Kirchseeon, Germany), which in turn were connected to a PC Polygraph HR (Synectics Medical, Stockholm, Sweden). The manometric signals were stored in a computer. During the manometric recordings, a minimally compliant pneumohydraulic pump (JS Biomedicals, Ventura CA, USA) perfused distilled water at 0.5 ml/min through each lumen.

All individuals were studied in the supine position after 12 hr of fasting. The catheter was introduced through the nose. For the study of proximal and distal esophageal contractions, the catheter was positioned with the proximal opening located 2 cm below the UES and the distal opening located 22 cm from the UES. Five swallows of a 5-ml bolus of water at room temperature alternated with five dry swallows were performed with an interval of at least 20 seconds between swallows.

Using the computer Polygram Upper GI software version 6.4 (Gastrosoft, Stockholm, Sweden), we measured the amplitude, duration, area under the curve (AUC) of contractions, and the time between the upstroke of contractions measured at 2 cm (proximal) and 22 cm (distal) from the UES. The AUC of the contractions represented amplitude x duration.

The statistical analysis was done by the Center of Quantitative Analysis of the Medical School of Ribeirao Preto USP (CEMEQ) using a linear model with mixed effects [19]. The model was adjusted using the Proc Mixed feature of the SAS software version 9 (SAS Institute, Cary, NC, USA) [20], as previously described [21]. The results were reported as mean and standard deviation, unless otherwise stated. The differences were considered significant when p < 0.05 in a two-tailed statistical analysis.

## Results

**Figure 1:**
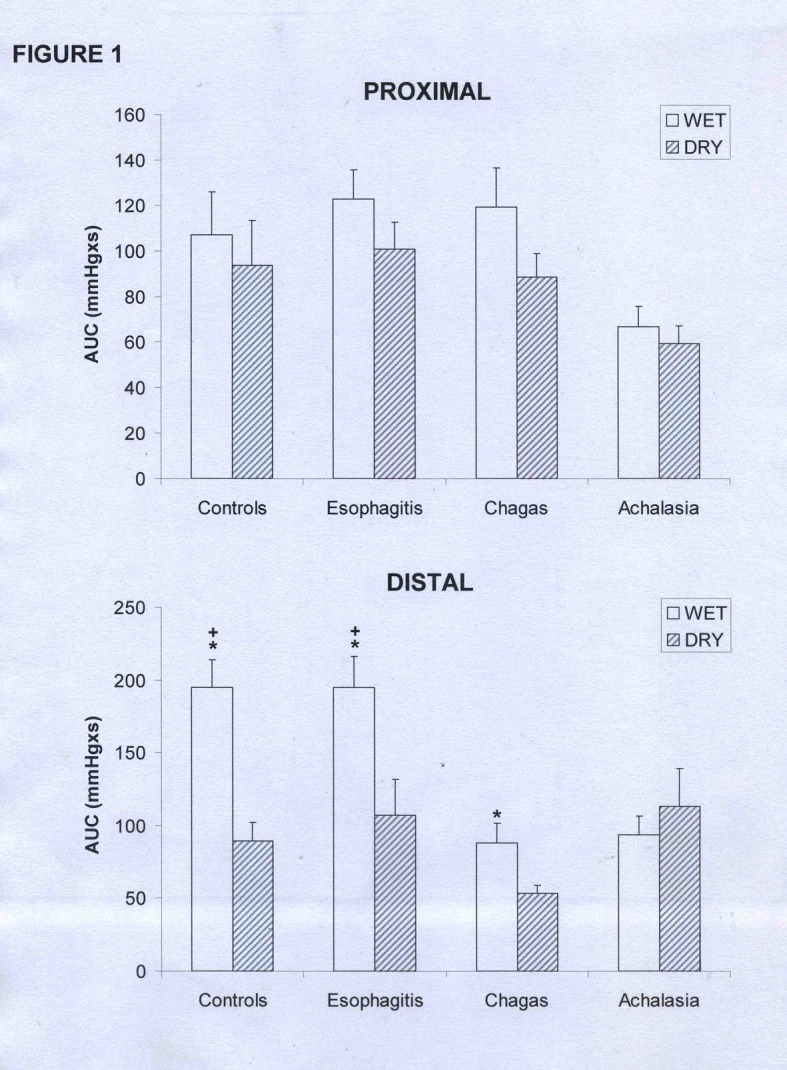
Area under the curve (AUC) of the proximal and distal esophageal contractions in patients with esophagitis (n = 28), Chagas’ disease (n = 37), idiopathic achalasia (n = 8), and controls (n = 31). (mean and SEM). *p < 0.02 vs dry, +p < 0.01 vs Chagas and achalasia.

In the proximal esophagus there was no difference in amplitude (Table 1), duration (Table 2) or AUC (Fig. 1) of contractions between wet and dry swallows in control subjects and in patients with esophagitis, achalasia and Chagas’ disease (p > 0.12).

**Table 1:** Amplitude of Esophageal Contraction (mmHg) measured in the Proximal and Distal Esophageal Body After Wet and Dry Swallows

	Proximal			Distal		
	Wet	Dry	p	Wet	Dry	p
Controls	95.1 (56.0)	78.7 (46.8)	0.22	88.3 (37.2)	45.0 (28.4)	< 0.01
Esophagitis	85.6 (34.6)	75.3 (39.7)	0.30	90.9 (42.2)	44.6 (32.6)	< 0.01
Achalasia	31.4 (11.0)^a^	32.4 (18.3)^a^	0.90	38.9 (16.0)^b^	46.1 (34.0)	0.60
Chagas	75.4 (49.3)	63.8 (40.4)	0.27	37.2 (34.6)^b^	24.7 (15.4)^c^	0.05

Esophagitis (n = 28), Idiopathic Achalasia (n = 8), Chagas’ Disease (n = 37), and Controls (n = 31).

The results are reported as mean (SD). ^a^p < 0.02 vs esophagitis, Chagas, controls; ^b^p < 0.01 vs esophagitis, controls; ^c^p < 0.01 vs esophagitis, achalasia, controls

**Table 2:** Duration of Esophageal Contractions (Seconds) Measured in the Proximal and Distal Esophageal Body After Wet and Dry Swallows

	Proximal			Distal		
	Wet	Dry	p	Wet	Dry	p
Controls	2.5 (0.7)	2.6 (0.9)	0.75	3.9 (0.8)	3.3 (0.8)	< 0.01
Esophagitis	2.8 (0.7)	2.8 (0.7)	0.30	3.9 (1.1)	3.5 (1.6)	0.95
Achalasia	3.5 (1.1)^a^	3.5 (1.4)^a^	0.91	4.1 (1.1)	4.1 (1.0)	0.27
Chagas	3.1 (1.1)^a^	2.9 (0.8)	0.30	3.7 (1.0)	3.4 (0.8)	0.21

Esophagitis (n = 28), Idiopathic Achalasia (n = 8), Chagas’ Disease (n = 37), and Controls (n = 31). The results are reported as mean (SD). ^a^p < 0.05 vs esophagitis, controls.

With wet swallows the contractions in the distal esophagus had greater amplitude than with dry swallows in controls and in patients with esophagitis (Table 1, p < 0.01). There was no difference in patients with achalasia, and in patients with Chagas’ disease, with the results reaching borderline significance (p = 0.05).

In distal esophageal body the contractions of the controls had longer duration with wet swallows than with dry swallows (Table 2, p < 0.01), with no differences in patients with esophagitis, achalasia or Chagas’ disease (p > 0.20). The AUC for the distal esophagus was greater with wet swallows than with dry swallows in controls, patients with esophagitis and patients with Chagas’ disease (Fig. 1, p < 0.02).

**Figure 2:**
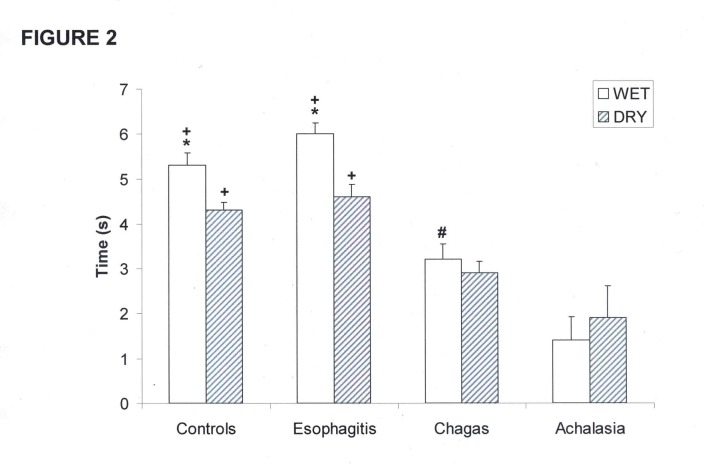
Interval between the upstroke of proximal and distal esophageal contractions in patients with esophagitis (n = 28), Chagas’ disease (n = 37), idiopathic achalasia (n = 8), and controls (n = 31). (mean and SEM). *p < 0.01 vs dry, +p < 0.01 vs Chagas and achalasia, #p < 0.01 vs achalasia.

The interval between the upstroke of the proximal and distal contractions was longer with wet swallows than with dry swallows in controls and in patients with esophagitis (Fig. 2, p < 0.01). This interval was shorter in patients with achalasia and Chagas’ disease compared with controls and patients with esophagitis, with no difference between wet and dry swallows.

Patients with achalasia had a low contraction amplitude in the proximal esophagus than patients with esophagitis, Chagas’ disease and controls, after both wet and dry swallows (Table 1, p < 0.02). In the distal esophagus wet swallows cause lower contraction amplitude in patients with achalasia and Chagas’ disease than in patients with esophagitis and controls (Table 1, p < 0.01).

The duration of contraction in the proximal esophagus was longer in patients with achalasia than in patients with esophagitis and controls (Table 2, p < 0.05), with no differences between groups in the distal esophagus.

There was no difference between groups in the AUC of the proximal esophagus (Fig. 1, p > 0.08). In the distal esophagus, wet swallows caused a lower AUC in patients with Chagas’ disease and achalasia than in patients with esophagitis and controls (p < 0.01).

The interval between the upstroke of proximal and distal esophageal contraction, with wet and dry swallows, was longer in patients with esophagitis and controls than in patients with Chagas’ disease and achalasia (Fig. 2, p < 0.01)

## Discussion

The esophageal phase of swallowing begins when the bolus reaches the proximal esophageal body and continues until the bolus crosses the LES. It is independent of both the oral and pharyngeal phases. The peristaltic movement in the esophageal body is consequent to the presence of the bolus inside the proximal esophagus, which stimulates the esophagus to contract following a swallow of solid, liquid or air boluses. The initiation of the esophageal phase of swallowing is activated by the presence of the bolus inside the esophagus and is strongly dependent upon feedback from the esophagus [16].

We did not find differences between wet and dry swallows in the proximal esophagus, suggesting a central control of the response to swallows which causes a proximal esophageal response that is not dependent on the kind of the bolus. However, patients with achalasia had a lower contraction amplitude and longer contraction duration than controls and patients with esophagitis. Since in Chagas’ disease and achalasia there is no clear demonstration of impairment of the premotor neurons of the nucleus tractus solitarius or of the motor nuclei of the dorsal motor nucleus and the dorsal nucleus ambiguus, the alterations of contractions seen in the proximal esophagus should be consequent to impairment of local innervation. In the chronic phase of Chagas’ disease there are no major neurologic deficits or dysfunction [22] that could explain the alterations of esophageal motility, but there is loss of neurons in the esophageal myenteric plexus [5, 6]. In achalasia there are alterations of the esophageal myenteric plexus [7, 8], with the possibility, not completely clear yet, of some alterations of the central control of swallowing [8, 23].

In the distal esophagus, wet swallows caused a more intense contraction than dry swallows in patients with Chagas’ disease, esophagitis and controls, but not in patients with achalasia. Previous publications have shown that dry swallows cause a different esophageal response than wet swallows in healthy subjects [2, 3, 17, 24]. This response is modulated by a cholinergic neural excitatory input [24], which is, at least partially, impaired in Chagas’ disease and achalasia [5-7, 23, 25]. Thus, patients with Chagas’ disease should not have differences in esophageal contractions with wet or dry swallows. However, in the present investigation we found that patients with Chagas’ disease had lower contraction amplitude and AUC in the distal esophagus after dry swallows compared with wet swallows, with borderline statistical significance. The esophageal involvement by Chagas’ disease has a wide spectrum, a fact that causes a significant variation of clinical esophageal manifestations [11, 12]. In a previous publication [17], the AUC measured at 17 cm from the UES in Chagas’ disease patients fell from 93.0 (67.9) mmHg with a 5 ml bolus of water to 80.5 (69.6) mmHg with dry swallows. But this difference was not significant (p > 0.05). In the present study the AUC measured at 22 cm from the UES fell from 88.1 (81.7) mmHg after wet swallows to 53.6 (33.3) mmHg after dry swallows (p = 0.02). The different esophageal involvement by the disease may explain the different conclusion observed in the various groups studied and may be due to the fact that this involvement is not of the same intensity in all patients, as is seen in patients with idiopathic achalasia, for whom the alterations in esophageal motility are a condition for diagnosis.

The time of propagation of the esophageal contraction from the proximal to the distal esophagus was shorter with dry than with wet swallows in patients with esophagitis and controls, but not in patients with Chagas’ disease and achalasia. Patients with impairment of esophageal innervation caused by achalasia or Chagas’ disease lose the inhibitory esophageal innervation, and the esophageal body responds to the contraction stimulus in an abnormal way, most of the time with simultaneous contraction throughout the esophageal body [9, 10], with wet or dry swallows.

Comparison of proximal contractions between the groups revealed a lower contraction amplitude in patients with achalasia and a longer contraction duration in patients with achalasia and Chagas’ disease. Previous results have shown that the amplitude of proximal esophageal contractions is similar in patients with Chagas’ disease and controls [25-28], with one study reporting lower contraction amplitude in Chagas’ disease [29]. There are also results showing an increase in the duration of proximal contractions [28]. Investigations performed in achalasia have found lower than normal proximal contraction amplitude [25-27, 30].

In the distal esophagus the contractions have low amplitude in Chagas’ disease [9, 11, 25] and achalasia [8-10, 25]. The results obtained here were as expected for these diseases, which involve a significant loss of neurons of the myenteric plexus as the more outstanding histopathologic finding.

The patients with esophagitis did not have alterations of esophageal contractions compared to control subjects. Ineffective esophageal motility [31] can predispose the esophageal mucosa to the effect of gastroesophageal reflux, but there is controversy about whether gastroesophageal reflux disease is associated with this alteration of esophageal motility [14, 15]. Alterations of esophageal motility are not a frequent observation in patients with esophagitis, most of the time being seen only in patients with a more intense esophagitis [13].

We performed five wet and five dry swallows because in a previous investigation we found that this number of swallows is sufficient for the evaluation of contraction amplitude and duration in normal subjects, patients with achalasia and patients with Chagas’ disease [32].

We conclude that the impairment of esophageal innervation decreases the contraction amplitude in the distal esophageal body, without differences between wet and dry swallows. In the proximal esophagus the alterations in esophageal physiology seen in idiopathic achalasia cause a decrease in esophageal contraction.
